# Non-Alcoholic Fatty Liver Disease in Long-Term Type 2 Diabetes: Role of rs738409 *PNPLA3* and rs499765 *FGF21* Polymorphisms and Serum Biomarkers

**DOI:** 10.3390/molecules27103193

**Published:** 2022-05-17

**Authors:** Mauy Frujuello Mana, Maria Cândida R. Parisi, Maria Lucia Correa-Giannella, Arnaldo Moura Neto, Ademar Yamanaka, Marlone Cunha-Silva, Ana Mercedes Cavaleiro, Cristina Rodrigues dos Santos, Célia Regina Pavan, Tiago Sevá-Pereira, Sergio S. J. Dertkigil, Daniel F. Mazo

**Affiliations:** 1 Division of Gastroenterology (Gastrocentro), Department of Internal Medicine, School of Medical Sciences, University of Campinas (UNICAMP), Campinas 13083-878, SP, Brazil; mauymana@gmail.com (M.F.M.); yamanaka@unicamp.br (A.Y.); marlone.cunha@hotmail.com (M.C.-S.); stin@unicamp.br (C.R.d.S.); cpavan@unicamp.br (C.R.P.); tiagoseva@gmail.com (T.S.-P.); 2 Division of Endocrinology, Department of Internal Medicine, School of Medical Sciences, University of Campinas (UNICAMP), Campinas 13083-887, SP, Brazil; candidap@unicamp.br (M.C.R.P.); arnaldo.mouraneto@gmail.com (A.M.N.); 3 Laboratório de Carboidratos e Radioimunoensaios (LIM-18), Hospital das Clínicas HCFMUSP, Faculdade de Medicina, Universidade de São Paulo, Sao Paulo 01246-903, SP, Brazil; maria.giannella@fm.usp.br (M.L.C.-G.); anamercedes@usp.br (A.M.C.); 4 Department of Radiology, School of Medical Sciences, University of Campinas (UNICAMP), Campinas 13083-887, SP, Brazil; sdertkigil@fcm.unicamp.br; 5 Division of Clinical Gastroenterology and Hepatology, Department of Gastroenterology, University of São Paulo School of Medicine (FMUSP), Sao Paulo 05403-900, SP, Brazil

**Keywords:** non-alcoholic fatty liver disease, type 2 diabetes, liver fibrosis, genetic polymorphisms, biomarkers, elastography

## Abstract

Fibroblast growth factor 21 (FGF21) signaling and genetic factors are involved in non-alcoholic fatty liver disease (NAFLD) pathogenesis. However, these factors have rarely been studied in type 2 diabetes mellitus (T2D) patients from admixed populations such as in those of Brazil. Therefore, we aimed to evaluate rs738409 patanin-like phospholipase domain-containing protein (*PNPLA3*) and rs499765 *FGF21* polymorphisms in T2D, and their association with NAFLD, liver fibrosis, and serum biomarkers (FGF21 and cytokeratin 18 levels). A total of 158 patients were included, and the frequency of NAFLD was 88.6%, which was independently associated with elevated body mass index. Significant liver fibrosis (≥F2) was detected by transient elastography (TE) in 26.8% of NAFLD patients, and was independently associated with obesity, low density lipoprotein, and gamma-glutamyl transferase (GGT). *PNPLA3* GG genotype and GGT were independently associated with cirrhosis. *PNPLA3* GG genotype patients had higher GGT and AST levels; *PNPLA3* GG carriers had higher TE values than CG patients, and *FGF21* CG genotype patients showed lower gamma-GT values than CC patients. No differences were found in serum values of FGF21 and CK18 in relation to the presence of NAFLD or liver fibrosis. The proportion of NAFLD patients with liver fibrosis was relevant in the present admixed T2D population, and was associated with *PNPLA3* polymorphisms.

## 1. Introduction

Non-alcoholic fatty liver disease (NAFLD) is one of the most common liver diseases worldwide, affecting 25% of the general population [1]. In Latin America, the estimated prevalence of NAFLD (31%) is even higher than the worldwide rate [1]. The increasing prevalence of fatty liver is closely related to the global growth of type 2 diabetes mellitus (T2D) and obesity, two major drivers of the disease severity and progression [2,3]. Indeed, in T2D patients, the prevalence of NAFLD may be more than two times higher than in the general population, and advanced liver fibrosis may affect 17% of these patients, making this group of special interest for NAFLD evaluation [4,5].

Altered fibroblast growth factor 21 (FGF21) signaling has been implicated in NAFLD pathophysiology [6]. FGF21 is a hormone expressed in liver, pancreatic, and adipose tissues, and it is involved in energy homeostasis, insulin sensitivity, and glucose and lipid metabolism [7]. Studies in humans and animals have shown that FGF21 causes an increase in peripheral insulin sensitivity and stimulates glucose uptake and lipid oxidation via an increase in adiponectin [7,8]. Paradoxically, the circulating levels of FGF21 are elevated in patients with NAFLD, suggesting a state of resistance to FGF21 [8,9]. Exogenous FGF21 administration may overcome this resistance and become a potential pharmacological target for NAFLD management [10].

However, NAFLD has a multifactorial pathophysiology, and despite environmental and metabolic components, genetic factors may also play an important role in NAFLD development and progression [11,12]. Genetic variants in the patatin-like phospholipase domain-containing 3 (*PNPLA3*) gene are among the most studied genetic factors associated with NAFLD, and are one of the main determinants of inter-individual differences that are related to ethnic influence on liver fat content [3,13]. These variants have also been implicated in NAFLD occurrence and severity in admixed populations, such as that in Brazil [14]. However, a minimal amount of data concerning Brazilian T2D patients can be found [15]. The single nucleotide polymorphism (SNP) rs499765 C > G in the *FGF21* gene was found to be associated with serum FGF21 levels and NAFLD in a Chinese nondiabetic population [16], but no data in T2D patients with NAFLD are currently available.

In this context, the aim of this study was to evaluate two parameters: (1) the prevalence of, and factors associated with, NAFLD and liver fibrosis in a cohort of patients with T2D followed at an outpatient tertiary university center, and (2) the prevalence of SNPs rs738409 in *PNPLA3* and rs499765 in *FGF21* and their association with NAFLD, liver fibrosis, and serum FGF21 and cytokeratin 18 (CK18) levels in this population.

## 2. Materials and Methods

### 2.1. Clinical Design and Patients’ Selection

This study was designed as single-center longitudinal study conducted in patients with T2D who were followed between 2018 and 2020 at the Outpatient Units of the Endocrinology and Gastroenterology Division (Gastrocentro) of the University of Campinas (UNICAMP), Campinas, Brazil. Patients were invited to participate in the study and evaluated regarding the presence of NAFLD through documentation of liver steatosis on an abdominal ultrasound (US) and clinical and laboratory workups for the exclusion of other chronic liver diseases, such as alcoholic liver disease, hepatitis B and C, autoimmune hepatitis, hemochromatosis, alpha 1-antitrypsin deficiency, and Wilson’s disease.

The inclusion criteria were age 18–75 years old and presence of T2D. The exclusion criteria consisted of any other chronic liver disease, previous or current significant alcohol intake (>20 g/day for women and >30 g/day for men), human immunodeficiency virus (HIV), history of exposure to drugs associated with fatty liver (corticosteroids, amiodarone, methotrexate, and/or tamoxifen), previous liver transplantation, presence of other forms of diabetes, presence of relevant systemic diseases that could interfere with liver evaluation, and/or refusal to participate in the study. Patients who were included underwent blood sampling for biochemical analysis, evaluations of SNPs in *PNPLA3* and *FGF21* genes, and serum biomarkers (CK18 and FGF21). Additionally, the subjects underwent a liver US exam and were non-invasively evaluated for liver fibrosis based on FibroScan^®^.

### 2.2. Variables

Demographic and anthropometric data (age, gender, abdominal circumference, and body mass index (BMI)) were obtained, in addition to that regarding the presence of comorbidities (arterial hypertension, dyslipidemia, obesity, coronary arterial disease (CAD), hypothyroidism, chronic kidney disease (CKD), and tobacco use). The presence of CAD was defined by review of medical charts, which evaluated clinical history, electrocardiographic criteria, and/or an ischemic stress test showing CAD, description of angina, and/or previous acute myocardial infarction. CKD was defined as a reduction in glomerular filtration rate to values <60 mL/min/1.73 m^2^ for ≥3 months. Regarding T2D, time since diagnosis (>10 years was defined as long-term), medications used, and related complications (retinopathy, diabetic kidney disease and neuropathy) were also recorded.

Serum biochemistry included alanine aminotransferase (ALT), aspartate aminotransferase (AST), gamma-glutamyl transferase (GGT), creatinine, ferritin, albumin, platelet count, glycated hemoglobin (HbA1C), total cholesterol, high- and low-density lipoproteins (HDL and LDL, respectively), and triglycerides. These values were collected after a 12 h overnight fasting period.

### 2.3. Definition of Non-Alcoholic Fatty Liver Disease

NAFLD was defined when the liver US (Toshiba Aplio, Toshiba America Medical Systems Inc., Tustin, CA, USA) indicated hepatic steatosis and when other chronic liver diseases, significant alcohol consumption, and/or steatogenic medications were excluded. Patients who had a pattern of chronic liver disease or cirrhosis on US liver evaluation [17] in the absence of other etiology to explain the liver disease were also classified as having NAFLD.

### 2.4. Non-Invasive Evaluation of Liver Fibrosis

Transient elastography (TE) by FibroScan^®^ (Model 402, Echosens, Paris, France) was performed in the NAFLD patients. The test result was considered reliable when the success rate was above 60% with an interquartile range (IQR) < 30% of the median value of the measurements (IQR/median ≤ 0.3). Fibrosis staging was classified according to liver stiffness measurement (LSM) results: (1) significant hepatic fibrosis (F ≥ 2): LSM ≥ 7.0 kPa; (2) significant/advanced fibrosis (F2–F3): LSM ≥ 7 kPa and ≤ 10.3 kPa; and (3) cirrhosis (F4): LSM > 10.3 kPa [18].

### 2.5. Serum Biomarkers

For analysis of M65 CK18 and FGF21 levels, blood samples were collected in dry tubes, and serum was stored at −80 °C until analysis. Commercial enzyme-linked immunosorbent assay (ELISA) kits from Finetest EH2820 and EH0130 (Wuhan Fine Biotech Co., Ltd., Wuhan, China) were used for the evaluation of CK18 (measured in ng/mL) and FGF21 (measured in pg/mL), respectively, as per the manufacturers’ recommendations.

### 2.6. DNA Extraction and Genotyping

Genomic DNA was extracted using the salting-out technique from blood samples collected in EDTA tubes. DNA quantification was determined by spectrophotometry (GeneQuant DNA/RNA Calculator, Pharmacia, LKC Biotechnology, Uppsala, Sweden). After DNA extraction from leucocytes, the samples were stored at −20 °C until evaluation of the polymorphisms. Genotyping of the SNPs rs738409 in *PNPLA3* and rs499765 in *FGF21* was performed by real-time polymerase chain reaction (Applied Biosystems, ThermoFisher Brand, Foster City, CA, USA) using the TaqMan^TM^ SNP Genotyping Assay, human/*PNPLA3* (C_7241_10) and TaqMan^TM^ SNP Genotyping Assay, human/*FGF21* (C_987279_10) (ThermoFisher, Foster City, CA, USA), respectively, as per the manufacturer’s recommendations. The equipment used for reading was Step One Plus from Applied Biosystems.

### 2.7. Ethical Considerations

This study was approved by the Ethics Committee of UNICAMP (Approval No. 2,766,627). The protocol was conducted in accord with the ethical guidelines of the 2013 World Medical Association Declaration of Helsinki [19]. Informed consent was obtained from participants prior to starting the study.

### 2.8. Statistical Analysis

To analyze the demographic, clinical, and biochemical variables, we obtained the frequencies (absolute and percentage) of the categorical variables and descriptive measures (mean, standard deviation, median, and minimum and maximum values) for the quantitative variables. To assess the association between categorical variables, the chi-squared test was used, and when necessary, Fisher’s exact test. For numerical variables, the Mann–Whitney and Kruskal–Wallis tests were used. Univariate and multivariate logistic regression were performed to assess factors related to the presence of NAFLD, NAFLD with significant fibrosis (≥F2), and cirrhosis (F4), according to TE measurement. A stepwise method of variable selection was used in the multivariate logistic regression analysis. Odds ratio (OR) measures with 95% confidence intervals (95% CI) were obtained. A probability value of <0.05 was considered significant. Regarding the genetic polymorphisms, the Hardy–Weinberg equilibrium was evaluated. Heterozygosity and the polymorphic information content (PIC) were also calculated. The Statistical Analysis System (SAS) for Windows software package, version 9.4 (SAS Institute Inc., 2002–2008, Cary, NC, USA) was used for the statistical analyses conducted by a biomedical statistician from the Statistics Service at the School of Medical Sciences of UNICAMP.

## 3. Results

**Figure 1:**
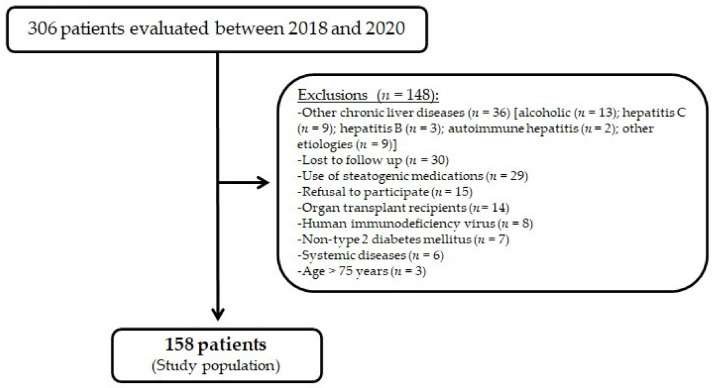
Flowchart of study population enrollment.

A total of 306 patients were evaluated for study inclusion. Of these, 148 patients did not meet the inclusion criteria or became ineligible during the study evaluation, as shown in Figure 1. The study population consisted of 158 patients, among whom 62% (98) were women. The mean age was 61.2 years, and the mean duration of diabetes was 18.2 years. Regarding comorbidities, 90.5% (143) had hypertension, 83.5% (132) had dyslipidemia, 24.7% (39) had CAD, and 20.9% (33) had CKD. Regarding T2D complications, 60.8% (96) had retinopathy, 38.6% (61) had neuropathy, and 52.5% (83) had diabetic kidney disease. Most patients (80%, 127) were taking insulin, and their median HbA1C was 8.10%. The median waist circumference was 99 cm, and median BMI was 30.25 kg/m^2^, with 84.8% (134) of the patients presenting as overweight or obese; 81.6% (129) of the patients were taking statins. On laboratory evaluation, a minority of the patients had elevated aminotransferases levels (12.7% (20) for ALT >35 U/L and 5.7% (9) for AST >35 U/L). The main characteristics of the study patients are detailed in Table 1.

### 3.1. Evaluation of NAFLD and Liver Fibrosis

On liver US, 75% (118) of the participants had steatosis (16.1% (19) mild, 45.8% (54) moderate, and 38.1% (45) severe), 11% (17) had signs of chronic liver disease, and five patients (3%) presented with a cirrhosis pattern. Thus, the prevalence of NAFLD was 88.6% (140/158). The mean LSM result based on TE was 7.13 ± 7.02 kPa, and 17.3% (22/127) of NAFLD patients presented results suggestive of significant/advanced fibrosis (F2–F3), while 9.4% (12/127) of the patients were classified by TE as having cirrhosis. Technical difficulties due to central adiposity and obesity were responsible for two unreliable TE results, which were excluded from the analysis.

### 3.2. Factors Associated with NAFLD

Based on an age-adjusted univariate logistic regression analysis, the factors associated with the presence of NAFLD were obesity (OR: 4.6; 95% CI: 1.4–15.1; *p* = 0.0112) and elevated BMI (*p* = 0.0019): (1) overweight (OR: 10.8; 95% CI: 2.5–46.6), (2) grade I obesity (OR: 10.4; 95% CI: 2.4–44.4), or (3) grade II or III obesity (OR: 15.1; 95% CI: 1.7–133.8). The only variable independently associated with the presence of NAFLD was elevated BMI (*p* = 0.0024): (1) overweight (OR: 22.6; 95% CI: 2.4–206.4), (2) grade I obesity (OR: 20.0; 95% CI: 2.1–184.4), or (3) obesity grade II or III (OR: 12.6; 95% CI: 1.3–119.0). These findings are detailed in Table 2. No differences were found in serum values of FGF21 and CK18 biomarkers in regard to the presence of NAFLD. In patients with or without NAFLD, the median FGF21 serum values were 387.2 versus 358.3 pg/mL (*p* = 0.1104); and for CK18, 0.7 versus 0.8 ng/mL (*p* = 0.7492).

### 3.3. Factors Associated with Significant Liver Fibrosis and Cirrhosis in NAFLD Patients

In the association tests, those with significant liver fibrosis based on the TE results, compared to patients without fibrosis, had higher serum ferritin levels (228.1 ± 222.7 versus 155.9 ± 162.5 ng/mL; *p* = 0.0242), and higher values of GGT (70.2 ± 57.1 versus 35.8 ± 25.0 U/L; *p* = 0.0003), ALT (29.5 ± 28.7 versus 20.5 ± 11.5 U/L; *p* = 0.0045), and AST (26.3 ± 18.9 versus 18.7 ± 5.4 U/L; *p* = 0.125). Obesity was also more frequent in patients with significant liver fibrosis (76.5% versus 50.5%; *p* = 0.0089), as were values above the upper limit of normality for AST (17.6% versus 2.2%; *p* = 0.0047) and ALT (23.5% versus 8.6%; *p* = 0.0348). The presence of the G allele of SNP rs738409 in *PNPLA3* gene was also associated with significant liver fibrosis (*p* = 0.0184). In the age-adjusted univariate logistic regression analysis, the factors associated with significant liver fibrosis were obesity (OR: 3.0; 95% CI: 1.2–7.5; *p* = 0.0140); *PNPLA3* G/G genotype (OR: 5.0; 95% CI: 1.3–18.1; *p* = 0.0338); higher serum levels of GGT (OR: 1.021; 95% CI: 1.009–1.033; *p* = 0.0005) and AST (OR: 1.084; 95% CI: 1.020–1.151; *p* = 0.0091); and lower platelet count (OR: 0.993; 95% CI: 0.987–0.999; *p* = 0.0284). AST above the reference value (>35 U/L) also showed an association (OR: 8.4; 95% CI: 1.5–45.0; *p* = 0.0126). Obesity (OR: 2.8; 95% CI: 1.04–7.71; *p* = 0.0412), LDL (OR: 0.984; 95% CI: 0.970–0.998; *p* = 0.0212), and GGT (OR: 1.029; 95% CI: 1.013–1.044; *p* = 0.0002) were independently associated with significant liver fibrosis, as shown in Table 3.

In the age-adjusted univariate logistic regression analysis, the G/G genotype of the SNP rs738409 in *PNPLA3* was associated with cirrhosis (OR: 16.4; 95% CI: 3.1–85.8; *p* = 0.0010), as well as high serum levels of GGT (OR: 1.022; 95% CI: 1.009–1.035; *p* = 0.0006) and AST (OR: 1.050; 95% CI: 1.004–1.098; *p* = 0.0325), and lower platelet count (OR: 0.978; 95% CI: 0.966–0.991; *p* = 0.0009). Elevated aminotransferases were also associated with cirrhosis, as well as higher levels of ALT (OR: 5.0; 95% CI: 1.3–19.7; *p* = 0.0188) and AST (OR: 21.3; 95% CI: 4.0–112.2; *p* < 0.0003). In the multivariate analysis, GGT levels (OR: 1.022; 95% CI: 1.008–1.036; *p* = 0.0021) and the G/G genotype of the SNP rs738409 in *PNPLA3* (OR: 13.2; 95% CI: 2.2–77.5; *p* = 0.0040) were associated with cirrhosis. These results are described in Table 4.

### 3.4. PNPLA3 and FGF21 Polymorphisms

The distribution of the genotypes for both evaluated SNPs was consistent with the Hardy–Weinberg equilibrium. The heterozygosity coefficient and PIC values for the *PNPLA3* SNP were 0.5878 and 0.5013, respectively. For the *FGF21* SNP, these values were 0.6264 and 0.5494, respectively. The frequencies of the genotypes for both SNPs did not differ between patients with and without NAFLD, as shown in Table 5.

Patients with the G/G genotype of the SNP rs738409 in *PNPLA3* had higher GGT values compared to C/C and C/G genotypes (71.1 ± 57.2 versus 42.8 ± 42.3 and 38.0 ± 30.5 U/L, respectively; *p* = 0.0336), in addition to higher AST values (32.8 ± 24.7 versus 19.2 ± 6.9 and 19.3 ± 4.9 U/L; *p* = 0.0039). AST levels were elevated more frequently in individuals with the G/G genotype (25%) compared to other genotypes (*p* = 0.0079). In addition, GG carriers had higher TE values than CG carriers (*p* = 0.0154), as shown in Table 6.

Regarding SNP rs499765 in *FGF21*, patients with the CG genotype showed lower GGT values than those with the CC genotype (33 ± 28.4 versus 53.7 ± 41.2 U/L; *p* = 0.0013). No other associations with biochemical and clinical variables and non-invasive assessment of liver fibrosis were detected. There was no association of serum FGF21 and CK18 values with the genotypes of SNPs rs738409 in *PNPLA3* (*p* = 0.3416 and 0.4177, respectively) and rs499765 in *FGF21* (*p* = 0.5633 and 0.2403, respectively).

## 4. Discussion

The present study evaluated a long-term T2D population at a tertiary university center and found an 88.6% prevalence of NAFLD, among which more than a quarter of participants had significant liver fibrosis based on TE assessment. Elevated BMI was independently associated with fatty liver, and also with significant liver fibrosis, along with LDL and GGT levels. In contrast, cirrhosis was associated with GGT levels and with the GG genotype of the SNP rs738409 in *PNPLA* in the multivariate logistic regression analysis. The evaluated *PNPLA3* and *FGF21* SNPs were not associated with NAFLD among T2D patients, but the *PNPLA3* GG genotype carriers had higher GGT and AST values compared to the other genotypes, and higher TE LSM values than the CG carriers. Participants with the *FGF21* CG genotype showed lower GGT values than the CC genotype carriers.

T2D and NAFLD are strongly related. The pooled prevalence of NAFLD diagnosed by US in T2D patients in a recent systematic review and meta-analysis was 59.21%, and this increased to 91.62% when it was diagnosed by either liver biopsy, non-invasive markers, or radiologic methods [4]. Among 561 T2D American patients, 30% of them followed at endocrinology outpatient clinics, Lomonaco et al. (2021) reported a 70% prevalence of steatosis detected by controlled attenuation parameter elastography [5]. The higher NAFLD detection rate in the present study could be attributed to the cohort profile, composed exclusively from tertiary center outpatients with long-term T2D (mean of 18 years) and poor glycemic control (mean HbA1C of 8.5%). In addition, almost 85% of the patients were overweight or obese, a factor that was independently associated with NAFLD in the present study. Indeed, evidence from a meta-analysis of 21 cohort studies showed that obesity independently led to a 3.5-fold increase in the risk of developing NAFLD [20]. The predominant environmental metabolic conditions, such as obesity and long-term T2D with poor glycemic control, may have hindered an association between rs738409 in *PNPLA* and NAFLD in this population.

Liver fibrosis is a strong predictor of all-cause and liver-related mortality in NAFLD patients, even at moderate stages (F2) [21,22]. In the study population, significant liver fibrosis (≥F2), as evaluated by TE, was found in 26.7% of NAFLD patients, a value higher than the 15% reported by Lomonaco et al. (2021) [5]. In addition to the aforementioned population characteristics, this difference in the prevalence of significant liver fibrosis could be due to the lower LSM cut-off definition adopted in the present study (LSM ≥ 7.0 kPa). Advanced liver fibrosis assessed by liver biopsy is described in 17% of T2D patients with NAFLD [4]. Due to a higher proportion of patients in the intermediate category of serum non-invasive markers of fibrosis, such as NAFLD Fibrosis Score and FIB4 [23], and the possible suboptimal performance of these in T2D patients [24], some authors have suggested to first use TE in this context to rule out advanced fibrosis [25].

Along with obesity, higher GGT levels were also independently associated with significant liver fibrosis in the present cohort, as previously described [26,27]. GGT may be involved in the modulation of the redox equilibria within cells and neighborhood, and low antioxidant defenses are correlated with elevated GGT levels [28,29]. The reduced ability of the liver to synthesize LDL with fibrosis progression could justify the association of LDL levels with significant liver fibrosis in this population [30]. Moreover, measurement of serum lipids and metabolites may assist to unravel the metabolic changes during NAFLD liver fibrosis progression [31]. Elevated aminotransferases in the present cohort were uncommon, since only 12.7% had ALT and 5.7% had AST above the reference values; findings that were quite similar to those described by Lomonaco et al. (2021) of 10% and 6%, respectively [5]. On the other hand, in patients with NAFLD, the mean ALT values were higher in those with significant fibrosis, but still within normal range according to the laboratory reference. This finding may be associated with the concepts of “normal” and “healthy” serum ALT levels, which motivated a proposal to reduce the ALT normal laboratory cut-off points to <19 U/L in women and <30 U/L in men with chronic hepatitis C or NAFLD [32].

The GG genotype of the *PNPLA3* SNP rs738409 was independently associated with cirrhosis in NAFLD patients with T2D. This SNP is responsible for the largest proportion of the genetic predisposition related to NAFLD, and is related to the whole spectrum of the disease [11,33]. In admixed populations, such as in Brazil, this SNP was also related to liver fibrosis [14,34]. However, data exclusively in T2D patients are scarce in Brazil. Machado et al. (2019) evaluated T2D Brazilian patients and reported significant liver fibrosis (TE LSM ≥ 7.9 kPa) in 25.2% of the cases, and a GG genotype frequency in rs738409 of 12% [15]; these results were similar to the present study values of 26.8% and 10.8%, respectively. In addition, significant liver fibrosis was independently associated with the G allele of rs738409 [15]. As recently reported by Gabriel-Medina et al. (2022), if a genetic risk factor, such as *PNPLA3* rs738409, coexists with T2D, a major metabolic driver of NAFLD progression, advanced liver fibrosis may result even more frequently [35]. This SNP could be useful for personalized risk prediction of liver fibrosis in T2D subjects in the future. Accordingly, a recent large prospective UK Biobank study consisting of 22,812 T2D participants demonstrated that *PNPLA3* rs738409 was independently associated with an increased risk of severe liver disease during follow-up [36].

The clinical significance of the CG genotype in *FGF21* rs499765, which was associated with lower GGT values than the CC genotype, needs to be further elucidated since it is the first description of this SNP in Brazilian patients. The same finding is true for FGF21 serum levels in admixed populations. As only 12 patients with cirrhosis were included, the role of the serum biomarkers in this group remains unclear. The relationship between T2D and NAFLD is bidirectional, and prospective data have shown that NAFLD patients have a 2.19-fold increased risk of incident diabetes over time [37]. Interestingly, a recent Korean longitudinal study reported that *PNPLA3* SNP rs738409 may modulate the risk of incident T2D in subjects both with and without NAFLD [38].

The present study brings some limitations. Evaluation of the use of antidiabetics that could potentially have an effect on NAFLD, such as pioglitazone and sodium glucose co-transporter-2 inhibitors, was not performed in this population. The lack of liver histological analysis hampered the steatohepatitis evaluation and the assessment of its impact on the results. In addition, the absence of histological data impaired the performance and usefulness of serum M65 CK18 and FGF21, biomarkers used for discrimination of steatohepatitis in NAFLD [39]. On the other hand, the lack of liver histological analysis could be acceptable, as non-invasive liver fibrosis evaluation is current clinical practice and broadens the applicability of the study findings beyond specialized liver centers. It should be noted that there is a proposal to change the term NAFLD to metabolic dysfunction-associated fatty liver disease (MAFLD), which does not currently discriminate steatosis from steatohepatitis [40,41]. The sample size was moderate, but the study provides a portrait of T2D patients from a mixed-race population with long-term disease, with findings that mirror those from larger cohorts [5,27], and it also adds a genetic evaluation.

## 5. Conclusions

In conclusion, in this evaluation of an admixed T2D population, we found that NAFLD with liver fibrosis by TE was relevant and associated with SNP rs738409 in *PNPLA3*. More studies are expected to clarify the involvement of the *FGF21* rs499765 SNP and of serum FGF21 levels in T2D and NAFLD patients.

## Figures and Tables

**Table 1:** Demographic, clinical, and biochemical characteristics of the included patients (*n* = 158).

Characteristics	T2D Patients (*n* = 158) % (*n*) or Mean ± SD
Age (years)	61.2 ± 9.4
Men/Women	38% (60)/62% (98)
T2D time since diagnosis (years)	18.2 ± 9.4
Overweight (BMI > 25 and <30)	32.9% (52/158)
Obesity (BMI ≥ 30)	51.9% (82/158)
BMI (kg/m^2^)	30.9 ± 6
Waist circumference (cm)	98.9 ± 17.7
Chronic kidney disease	20.9% (33)
Dyslipidemia	83.5% (132)
Hypertension	90.5% (143)
Hypothyroidism	24.1% (38)
Tobacco use	7% (11)
Coronary artery disease	24.7% (39)
Diabetes complications	
Retinopathy	60.8% (96)
Diabetic kidney disease	52.5% (83)
Neuropathy	38.6% (61)
Glycated hemoglobin (%)	8.5 ± 1.9
Total cholesterol (mg/dL)	162.0 ± 47.0
LDL (mg/dL)	88.0 ± 38.0
HDL (mg/dL)	43.0 ± 18.0
Triglycerides (mg/dL)	160.0 ± 97.0
Ferritin (ng/mL)	177.0 ± 185.0
GGT (U/L)	44.0 ± 42.0
AST (U/L)	20.0 ± 10.0
ALT (U/L)	22.0 ± 17.0
Elevated AST levels (>35 U/L)	5.7% (9)
Elevated ALT levels (>35 U/L)	12.7% (20)
Creatinine (mg/dL)	1.3 ± 1.1
Albumin (g/dL)	4.0 ± 0.7
Platelets (×10^9^/L)	245.5 ± 70.4

ALT: Alanine aminotransferase; AST: Aspartate aminotransferase; BMI: Body mass index; GGT: Gamma glutamyl transferase; HDL: High-density lipoprotein; LDL: Low-density lipoprotein; SD: Standard deviation; T2D: Type 2 diabetes mellitus.

**Table 2:** Factors associated with NAFLD in patients with T2D (*n* = 158).

Variable	Univariate Analysis			Multivariate Analysis		
	OR	95% CI	*p*-Value	OR	95% CI	*p*-Value
Women	1.0	0.3–2.8	0.9654			
Diagnosis of T2D (years)	0.9	0.9–1.0	0.9785			
Overweight	10.8	2.5–46.6	0.0019 ^1^	22.6	2.4–206.4	0.0024 ^1^
Obesity Grade I	10.4	2.4–44.4		20.0	2.1–184.4	
Obesity Grade II or III	15.1	1.7–133.8		12.6	1.3–119.0	
Obesity (BMI ≥ 30)	4.6	1.4–15.1	0.0112 ^1^			
Waist circumference	1.0	0.9–1.0	0.0686			
Chronic kidney disease	1.0	0.3–3.2	0.9975			
Dyslipidemia	1.0	0.2–4.1	0.9222			
Hypothyroidism	0.8	0.2–2.6	0.7988			
Coronary artery disease	0.8	0.2–2.6	0.8002			
Diabetes complications						
Retinopathy	0.3	0.1–1.2	0.1074			
Diabetic kidney disease	0.3	0.1–1.1	0.0929			
Neuropathy	0.5	0.2–1.6	0.3250			
Glycated hemoglobin	0.9	0.7–1.2	0.7719			
Total cholesterol	1.0	0.9–1.0	0.3507			
LDL	1.0	0.9–1.0	0.7273			
Triglycerides	1.0	0.9–1.0	0.1446			
Ferritin	1.0	0.9–1.0	0.9616			
GGT	1.0	0.9–1.0	0.6894			
AST	1.0	0.9–1.1	0.4316			
ALT	1.0	0.9–1.0	0.9888			
Creatinine	0.8	0.5–1.1	0.2234			
Albumin	1.3	0.4–3.8	0.5423			
Platelet count	1.0	0.9–1.0	0.9592			
FGF21	1.0	0.9–1.0	0.0863			
CK18	0.9	0.7–0.9	0.1539			
*PNPLA3* GG genotype	1.0	0.1–10.4	0.5692			
*PNPLA3* CG genotype	2.0	0.5–7.9				
*FGF 21* GG genotype	1.2	0.2–6.9	0.9382			
*FGF 21* CG genotype	1.2	0.3–5.3				

Age-adjusted logistic regression analysis. ALT: Alanine aminotransferase; AST: Aspartate aminotransferase; BMI: Body mass index; CI: Confidence interval; CK18: Cytokeratin 18; FGF 21: Fibroblast growth factor 21; GGT: Gamma glutamyl transferase; HDL: High-density lipoprotein; LDL: Low-density lipoprotein; OR: Odds ratio; NAFLD: Non-alcoholic fatty liver disease; T2D: Type 2 diabetes mellitus. ^1^ *p*-value < 0.05.

**Table 3:** Factors associated with significant liver fibrosis in NAFLD patients with T2D (*n* = 127).

Variable	Univariate Analysis			Multivariate Analysis		
	OR	95% CI	*p*-Value	OR	95% CI	*p*-Value
Women	1.1	0.4–2.5	0.7957			
Diagnosis of T2D (years)	0.9	0.9–1.0	0.6359			
Overweight	0.7	0.1–3.3	0.4757			
Obesity Grade I	1.4	0.3–6.1				
Obesity Grade II	1.0	0.1–5.6				
Obesity Grade III	3.2	0.4–24.6				
Obesity (BMI ≥ 30)	3.0	1.2–7.5	0.0140 ^1^	2.837	1.043–7.719	0.0412 ^1^
Waist circumference	1.0	0.9–1.0	0.7117			
Chronic kidney disease	0.4	0.1–1.4	0.1667			
Dyslipidemia	0.5	0.1–1.3	0.1684			
Hypertension	0.7	0.2–2.6	0.7011			
Hypothyroidism	1.7	0.7–4.3	0.1938			
Tobacco use	0.7	0.1–3.6	0.6965			
Coronary artery disease	0.9	0.3–2.4	0.8658			
Diabetes complications						
Retinopathy	0.9	0.3–2.0	0.7840			
Diabetic kidney disease	0.5	0.2–1.2	0.1616			
Neuropathy	0.5	0.2–1.3	0.1946			
Glycated hemoglobin	0.9	0.7–1.1	0.7910			
Total cholesterol	0.9	0.9–1.0	0.3791			
LDL	0.9	0.9–1.0	0.2014	0.980	0.970–0.998	0.0212 ^1^
Triglycerides	1.0	0.9–1.0	0.8070			
Ferritin	1.0	1.000–1.004	0.0512			
GGT	1.02	1.009–1.033	0.0005 ^1^	1.029	1.013–1.044	0.0002 ^1^
AST	1.08	1.020–1.151	0.0091 ^1^			
ALT	1.02	0.9–1.05	0.0842			
Creatinine	0.6	0.3–1.2	0.1733			
Albumin	1.020	0.6–1.7	0.9412			
Platelet count	0.99	0.98–0.99	0.0284 ^1^			
FGF21	1.0	0.9–1.0	0.9850			
CK18	1.0	0.8–1.2	0.6583			
*PNPLA3* GG genotype	5.0	1.3–18.1	0.0338 ^1^			
*PNPLA3* CG genotype	2.1	0.8–5.2				
*FGF 21* GG genotype	2.5	0.8–7.7	0.2084			
*FGF 21* CG genotype	1.0	0.4–2.5				

Age-adjusted logistic regression analysis. ALT: Alanine aminotransferase; AST: Aspartate aminotransferase; BMI: Body mass index; CI: Confidence interval; CK18: Cytokeratin 18; FGF21: Fibroblast growth factor 21; GGT: Gamma glutamyl transferase; HDL: High-density lipoprotein; LDL: Low-density lipoprotein; OR: Odds ratio; NAFLD: Non-alcoholic fatty liver disease; T2D: Type 2 diabetes mellitus. ^1^ *p*-value < 0.05.

**Table 4:** Factors associated with cirrhosis in NAFLD patients with T2D (*n* = 127).

Variable	Univariate Analysis			Multivariate Analysis		
	OR	95% CI	*p*-Value	OR	95% CI	*p*-Value
Women	1.6	0.3–6.5	0.5065			
Diagnosis of T2D (years)	0.9	0.8–1.0	0.4137			
Overweight	0.8	0.08–8.79	0.7166			
Obesity Grade I	1.3	0.1–14.0				
Obesity Grade II	0.4	0.02–8.72				
Obesity Grade III	2.5	0.1–38.5				
Obesity (BMI ≥ 30)	2.2	0.5–8.8	0.2577			
Waist circumference	1.007	0.972–1.043	0.6965			
Chronic kidney disease	0.4	0.05–4.13	0.5078			
Dyslipidemia	0.5	0.1–2.2	0.4037			
Hypertension	2.3	0.2–21.2	0.4449			
Hypothyroidism	1.7	0.4–6.4	0.4074			
Coronary artery disease	0.9	0.1–4.8	0.9281			
Diabetes complications						
Retinopathy	0.6	0.1–2.2	0.5040			
Diabetic kidney disease	0.4	0.1–1.7	0.2421			
Neuropathy	0.8	0.1–3.4	0.7939			
Glycated hemoglobin	1.010	0.7–1.364	0.9457			
Total cholesterol	1.000	0.988–1.012	0.9682			
LDL	1.001	0.986–1.015	0.9235			
Triglycerides	0.999	0.992–1.005	0.6729			
Ferritin	1.000	0.997–1.004	0.9282			
GGT	1.022	1.009–1.035	0.0006 ^1^	1.022	1.008–1.036	0.0021 ^1^
AST	1.050	1.004–1.098	0.0325 ^1^			
ALT	1.0	0.993–1.037	0.1938			
Creatinine	0.9	0.4–1.8	0.8303			
Albumin	0.6	0.2–1.9	0.4143			
Platelet count	0.978	0.966–0.991	0.0009 ^1^			
FGF21	1.001	0.994–1.008	0.7396			
CK18	0.9	0.7–1.3	0.9908			
*PNPLA3* GG genotype	16.4	3.135–85.828	0.0010 ^1^	13.2	2.2–77.5	0.0040 ^1^
*PNPLA3* CG genotype	1.1	0.2–6.1				
*FGF 21* GG genotype	0.4	0.046–4.066	0.7628			
*FGF 21* CG genotype	0.8	0.2–3.0				

Age-adjusted logistic regression analysis. ALT: Alanine aminotransferase; AST: Aspartate aminotransferase; BMI: Body mass index; CI: Confidence interval; CK18: Cytokeratin 18; FGF 21: Fibroblast growth factor 21; GGT: Gamma glutamyl transferase; HDL: High-density lipoprotein; LDL: Low-density lipoprotein; OR: Odds ratio; NAFLD: Non-alcoholic fatty liver disease; T2D: Type 2 diabetes mellitus. ^1^ *p*-value < 0.05.

**Table 5:** Frequency of the single nucleotide polymorphisms in *PNPLA3* and in *FGF21*.

Genotype Frequency %/(*n*)					Total %	*p*-Value
		**C/C**	**C/G**	**G/G**		
***PNPLA3***	No NAFLD (*n* = 13)	61.5 (8)	30.8 (4)	7.7 (1)	100	0.6521
	NAFLD (*n* = 135)	48.1 (65)	40.8 (55)	11.1 (15)	100	
		**C/C**	**C/G**	**G/G**		
***FGF21***	No NAFLD (*n* = 13)	30.8 (4)	46.1 (6)	23.1 (3)	100	0.8660
	NAFLD (*n* = 135)	36.3 (49)	45.2 (61)	18.5 (25)	100	

Chi-squared and Fisher’s exact test. NAFLD: Non-Alcoholic Fatty Liver Disease.

**Table 6:** Demographic, clinical, and biochemical characteristics of the genotypes of the single nucleotide polymorphism in *PNPLA3* in patients with T2D (*n* = 148).

Characteristics	CC (*n* = 73) % (*n*) or Mean ± SD	CG (*n* = 59) % (*n*) or Mean ± SD	GG (*n* = 16) % (*n*) or Mean ± SD	*p*-Value
Age (years)	61.9 ± 8.3	61.3 ± 10.0	59.2 ± 8.5	0.5587
Men/Women	38.4% (28)/61.6% (45)	44.1% (26)/55.9% (33)	18.8% (3)/81.3% (13)	0.1819
T2D time since diagnosis (years)	18.9 ± 9.2	18.0 ± 9.9	18.3 ± 10.8	0.7235
Obesity (BMI ≥ 30 kg/m^2^)	53.4% (39)	59.3% (35)	62.5% (10)	0.7035
BMI (kg/m^2^)	31.0 ± 6.5	31.2 ± 5.3	29.6 ± 4.4	0.7218
Waist circumference (cm)	98.2 ± 16.9	98.5 ± 20.1	101.1 ± 13.3	0.7340
Chronic kidney disease	20.5% (15)	16.9% (10)	25.0% (4)	0.7405
Dyslipidemia	83.6% (61)	86.4% (51)	68.8% (11)	0.2433
Hypertension	94.5% (69)	84.7% (50)	87.5% (14)	0.1709
Hypothyroidism	24.7% (18)	22.0% (13)	31.3% (5)	0.7447
Tobacco use	6.8% (5)	8.5% (5)	0.0% (0)	0.6439
Coronary artery disease	20.5% (15)	27.1% (16)	25.0% (4)	0.6709
Diabetes complications				
Retinopathy	64.4% (47)	57.6% (34)	50% (8)	0.4991
Diabetic kidney disease	54.8% (40)	47.5% (28)	50% (8)	0.6990
Neuropathy	41.1% (30)	37.3% (22)	31.3% (5)	0.7410
Glycated hemoglobin (%)	8.5 ± 2.0	8.5 ± 1.8	8.2 ± 2.1	0.5623
Total cholesterol (mg/dL)	167.2 ± 51.4	155.6 ± 41.9	168.9 ± 54.2	0.2917
LDL (mg/dL)	94.0 ± 43.2	79.9 ± 30.9	90.7 ± 38.1	0.0958
HDL (mg/dL)	46.2 ± 23.9	39.3 ± 10.6	43.1 ± 14.6	0.1329
Triglycerides (mg/dL)	157.2 ± 95.6	157.6 ± 106.8	160.1 ± 67.2	0.7014
Ferritin (ng/mL)	164.3 ± 174.6	159.6 ± 154.2	208.1 ± 203.7	0.3577
GGT (U/L)	42.8 ± 42.3	38.0 ± 30.5	71.1 ± 57.2	0.0336 ^1^
AST (U/L)	19.2 ± 6.9	19.3 ± 4.9	32.8 ± 24.7	0.0039 ^1^
ALT (U/L)	21.5 ± 14.1	20.8 ± 7.8	36.6 ± 40.4	0.1083
Elevated AST levels (>35 U/L)	5.5% (4)	1.7% (1)	25.0% (4)	0.0079 ^1^
Elevated ALT levels (>35 U/L)	12.3% (9)	10.2% (6)	31.3% (5)	0.0838
Creatinine (mg/dL)	1.2 ± 0.6	1.2 ± 1.1	1.3 ± 0.9	0.3364
Albumin (g/dL)	4.1 ± 0.9	4.0 ± 0.4	3.9 ± 0.7	0.5519
Platelets (×10^9^/L)	247.9 ± 68.3	251.4 ± 68.4	197.8 ± 74.8	0.1000
Transient elastography (kPa)	6.0 ± 2.5 (*n* = 62)	6.8 ± 4.0 (*n* = 50)	14.2 ± 18.9 (*n* = 13)	0.0154 ^1^

Chi-squared, Kruskal–Wallis and Fisher’s exact test. For Dunn’s test: GGT: GG > C/C and CG; AST: GG> CC and CG; Transient elastography: GG > CG. ALT: Alanine aminotransferase; AST: Aspartate aminotransferase; BMI: Body mass index; GGT: Gamma glutamyl transferase; HDL: High-density lipoprotein; LDL: Low-density lipoprotein; SD: Standard deviation; T2D: Type 2 diabetes mellitus. ^1^ *p*-value < 0.05.

## Data Availability

The raw data required to reproduce these results are available from the corresponding author upon reasonable request.

## Sample Availability

The samples are available from the authors.

## References

[1] Pinto Marques Souza de Oliveira C., Pinchemel Cotrim H., Arrese M. (2019). Nonalcoholic Fatty Liver Disease Risk Factors in Latin American Populations: Current Scenario and Perspectives. Clin. Liver. Dis..

[2] Hossain N., Afendy A., Stepanova M., Nader F., Srishord M., Rafiq N., Goodman Z., Younossi Z. (2009). Independent predictors of fibrosis in patients with nonalcoholic fatty liver disease. Clin. Gastroenterol. Hepatol..

[3] Lu F.B., Hu E.D., Xu L.M., Chen L., Wu J.L., Li H., Chen D.Z., Chen Y.P. (2018). The relationship between obesity and the severity of non-alcoholic fatty liver disease: Systematic review and meta-analysis. Expert Rev. Gastroenterol. Hepatol..

[4] Younossi Z.M., Golabi P., de Avila L., Paik J.M., Srishord M., Fukui N., Qiu Y., Burns L., Afendy A., Nader F. (2019). The global epidemiology of NAFLD and NASH in patients with type 2 diabetes: A systematic review and meta-analysis. J. Hepatol..

[5] Lomonaco R., Godinez Leiva E., Bril F., Shrestha S., Mansour L., Budd J., Portillo Romero J., Schmidt S., Chang K.L., Samraj G. (2021). Advanced Liver Fibrosis Is Common in Patients with Type 2 Diabetes Followed in the Outpatient Setting: The Need for Systematic Screening. Diabetes Care.

[6] Tucker B., Li H., Long X., Rye K.A., Ong K.L. (2019). Fibroblast growth factor 21 in non-alcoholic fatty liver disease. Metabolism.

[7] Staiger H., Keuper M., Berti L., Hrabe de Angelis M., Häring H.U. (2017). Fibroblast Growth Factor 21-Metabolic Role in Mice and Men. Endocr. Rev..

[8] Hui X., Feng T., Liu Q., Gao Y., Xu A. (2016). The FGF21-adiponectin axis in controlling energy and vascular homeostasis. J. Mol. Cell. Biol..

[9] Barb D., Bril F., Kalavalapalli S., Cusi K. (2019). Plasma Fibroblast Growth Factor 21 Is Associated with Severity of Nonalcoholic Steatohepatitis in Patients with Obesity and Type 2 Diabetes. J. Clin. Endocrinol. Metab..

[10] Ritchie M., Hanouneh I.A., Noureddin M., Rolph T., Alkhouri N. (2020). Fibroblast growth factor (FGF)-21 based therapies: A magic bullet for nonalcoholic fatty liver disease (NAFLD)?. Expert Opin. Investig. Drugs.

[11] Martin K., Hatab A., Athwal V.S., Jokl E., Piper Hanley K. (2021). Genetic Contribution to Non-alcoholic Fatty Liver Disease and Prognostic Implications. Curr. Diab. Rep..

[12] Loomba R., Friedman S.L., Shulman G.I. (2021). Mechanisms and disease consequences of nonalcoholic fatty liver disease. Cell.

[13] Romeo S., Kozlitina J., Xing C., Pertsemlidis A., Cox D., Pennacchio L.A., Boerwinkle E., Cohen J.C., Hobbs H.H. (2008). Genetic variation in PNPLA3 confers susceptibility to nonalcoholic fatty liver disease. Nat. Genet..

[14] Mazo D.F., Malta F.M., Stefano J.T., Salles A.P.M., Gomes-Gouvea M.S., Nastri A.C.S., Almeida J.R., Pinho J.R.R., Carrilho F.J., Oliveira C.P. (2019). Validation of PNPLA3 polymorphisms as risk factor for NAFLD and liver fibrosis in an admixed population. Ann. Hepatol..

[15] Machado C.M., Leite N.C., França P.H., Cardoso C.R., Salles G.F., Villela-Nogueira C.A. (2019). PNPLA3 gene polymorphism in Brazilian patients with type 2 diabetes: A prognostic marker beyond liver disease?. Nutr. Metab. Cardiovasc. Dis..

[16] Jiang S., Zhang R., Li H., Fang Q., Jiang F., Hou X., Hu C., Jia W. (2014). The single nucleotide polymorphism rs499765 is associated with fibroblast growth factor 21 and nonalcoholic fatty liver disease in a Chinese population with normal glucose tolerance. J. Nutrigenet. Nutr..

[17] Simonovský V. (1999). The diagnosis of cirrhosis by high resolution ultrasound of the liver surface. Br. J. Radiol..

[18] European Association for Study of Liver, Asociacion Latinoamericana para el Estudio del Higado (2015). EASL-ALEH Clinical Practice Guidelines: Non-invasive tests for evaluation of liver disease severity and prognosis. J. Hepatol..

[19] World Medical Association (2013). World Medical Association Declaration of Helsinki: Ethical principles for medical research involving human subjects. JAMA.

[20] Li L., Liu D.W., Yan H.Y., Wang Z.Y., Zhao S.H., Wang B. (2016). Obesity is an independent risk factor for non-alcoholic fatty liver disease: Evidence from a meta-analysis of 21 cohort studies. Obes. Rev..

[21] Dulai P.S., Singh S., Patel J., Soni M., Prokop L.J., Younossi Z., Sebastiani G., Ekstedt M., Hagstrom H., Nasr P. (2017). Increased risk of mortality by fibrosis stage in nonalcoholic fatty liver disease: Systematic review and meta-analysis. Hepatology.

[22] Taylor R.S., Taylor R.J., Bayliss S., Hagström H., Nasr P., Schattenberg J.M., Ishigami M., Toyoda H., Wai-Sun Wong V., Peleg N. (2020). Association Between Fibrosis Stage and Outcomes of Patients with Nonalcoholic Fatty Liver Disease: A Systematic Review and Meta-Analysis. Gastroenterology.

[23] Patel P., Hossain F., Horsfall L.U., Banh X., Hayward K.L., Williams S., Johnson T., Bernard A., Brown N.N., Lampe G. (2018). A Pragmatic Approach Identifies a High Rate of Nonalcoholic Fatty Liver Disease with Advanced Fibrosis in Diabetes Clinics and At-Risk Populations in Primary Care. Hepatol. Commun..

[24] Bril F., McPhaul M.J., Caulfield M.P., Clark V.C., Soldevilla-Pico C., Firpi-Morell R.J., Lai J., Shiffman D., Rowland C.M., Cusi K. (2020). Performance of Plasma Biomarkers and Diagnostic Panels for Nonalcoholic Steatohepatitis and Advanced Fibrosis in Patients with Type 2 Diabetes. Diabetes Care.

[25] Castera L. (2020). Non-invasive tests for liver fibrosis in NAFLD: Creating pathways between primary healthcare and liver clinics. Liver Int..

[26] Korkmaz H., Unler G.K., Gokturk H.S., Schmidt W.E., Kebapcilar L. (2015). Noninvasive estimation of disease activity and liver fibrosis in nonalcoholic fatty liver disease using anthropometric and biochemical characteristics, including insulin, insulin resistance, and 13C-methionine breath test. Eur. J. Gastroenterol. Hepatol..

[27] Lai L.L., Wan Yusoff W.N.I., Vethakkan S.R., Nik Mustapha N.R., Mahadeva S., Chan W.K. (2019). Screening for non-alcoholic fatty liver disease in patients with type 2 diabetes mellitus using transient elastography. J. Gastroenterol. Hepatol..

[28] Koenig G., Seneff S. (2015). Gamma-Glutamyltransferase: A Predictive Biomarker of Cellular Antioxidant Inadequacy and Disease Risk. Dis. Markers.

[29] Corti A., Belcastro E., Dominici S., Maellaro E., Pompella A. (2020). The dark side of gamma-glutamyltransferase (GGT): Pathogenic effects of an 'antioxidant' enzyme. Free Radic. Biol. Med..

[30] Jaafar R.F., Hajj Ali A.M., Zaghal A.M., Kanso M., Habib S.G., Halaoui A.F., Daniel F., Mokaddem F., Khalife M.J., Mukherji D.M. (2019). Fibroscan and low-density lipoprotein as determinants of severe liver fibrosis in diabetic patients with nonalcoholic fatty liver disease. Eur. J. Gastroenterol. Hepatol..

[31] McGlinchey A.J., Govaere O., Geng D., Ratziu V., Allison M., Bousier J., Petta S., de Oliviera C., Bugianesi E., Schattenberg J.M. (2022). Metabolic signatures across the full spectrum of non-alcoholic fatty liver disease. JHEP Rep..

[32] Prati D., Taioli E., Zanella A., Della Torre E., Butelli S., Del Vecchio E., Vianello L., Zanuso F., Mozzi F., Milani S. (2002). Updated definitions of healthy ranges for serum alanine aminotransferase levels. Ann. Intern. Med..

[33] Anstee Q.M., Darlay R., Cockell S., Meroni M., Govaere O., Tiniakos D., Burt A.D., Bedossa P., Palmer J., Liu Y.L. (2020). Genome-wide association study of non-alcoholic fatty liver and steatohepatitis in a histologically characterised cohort. J. Hepatol..

[34] Lisboa Q.C., Nardelli M.J., Pereira P.A., Miranda D.M., Ribeiro S.N., Costa R.S.N., Versiani C.A., Vidigal P.V.T., Ferrari T.C.A., Couto C.A. (2020). PNPLA3 and TM6SF2 polymorphisms in Brazilian patients with nonalcoholic fatty liver disease. World J. Hepatol..

[35] Gabriel-Medina P., Ferrer-Costa R., Rodriguez-Frias F., Ciudin A., Augustin S., Rivera-Esteban J., Pericàs J.M., Selva D.M. (2022). Influence of Type 2 Diabetes in the Association of PNPLA3 rs738409 and TM6SF2 rs58542926 Polymorphisms in NASH Advanced Liver Fibrosis. Biomedicines.

[36] Tavaglione F., De Vincentis A., Jamialahmadi O., Pujia R., Spagnuolo R., Picardi A., Morano S., Valenti L., Romeo S., Vespasiani-Gentilucci U. (2021). Inborn and acquired risk factors for severe liver disease in Europeans with type 2 diabetes from the UK Biobank. JHEP Rep..

[37] Mantovani A., Petracca G., Beatrice G., Tilg H., Byrne C.D., Targher G. (2021). Non-alcoholic fatty liver disease and risk of incident diabetes mellitus: An updated meta-analysis of 501 022 adult individuals. Gut.

[38] Moon S., Chung G.E., Joo S.K., Park J.H., Chang M.S., Yoon J.W., Koo B.K., Kim W. (2022). A PNPLA3 Polymorphism Confers Lower Susceptibility to Incident Diabetes Mellitus in Subjects with Nonalcoholic Fatty Liver Disease. Clin. Gastroenterol. Hepatol..

[39] He L., Deng L., Zhang Q., Guo J., Zhou J., Song W., Yuan F. (2017). Diagnostic Value of CK-18, FGF-21, and Related Biomarker Panel in Nonalcoholic Fatty Liver Disease: A Systematic Review and Meta-Analysis. Biomed. Res. Int..

[40] Eslam M., Sanyal A.J., George J., International Consensus Panel (2020). MAFLD: A Consensus-Driven Proposed Nomenclature for Metabolic Associated Fatty Liver Disease. Gastroenterology.

[41] Eslam M., Newsome P.N., Sarin S.K., Anstee Q.M., Targher G., Romero-Gomez M., Zelber-Sagi S., Wai-Sun Wong V., Dufour J.F., Schattenberg J.M. (2020). A new definition for metabolic dysfunction-associated fatty liver disease: An international expert consensus statement. J Hepatol..

